# Effectiveness of a multifactorial intervention, consisting of self-management of antihypertensive medication, self-measurement of blood pressure, hypocaloric and low sodium diet, and physical exercise, in patients with uncontrolled hypertension taking 2 or more antihypertensive drugs: The MEDICHY study

**DOI:** 10.1097/MD.0000000000019769

**Published:** 2020-04-24

**Authors:** Fabián Unda Villafuerte, Joan Llobera Cànaves, Patricia Lorente Montalvo, María Lucía Moreno Sancho, Bartolomé Oliver Oliver, Patricia Bassante Flores, Andreu Estela Mantolan, Joan Pou Bordoy, Tomás Rodríguez Ruiz, Ana Requena Hernández, Alfonso Leiva, Matíes Torrent Quetglas, José María Coll Benejam, Pilar D’Agosto Forteza, Fernando Rigo Carratalà

**Affiliations:** a Coll D’en Rabassa Healthcare Centre; b Primary Care Research Unit of Mallorca, Balearic Health Services (IB-Salut); c Institut d’investigació Sanitària Illes Balears; d Santa Ponça Health Care Centre; e Son Cladera Healthcare Centre, Balearic Health Services (IB-Salut); f Spanish Society of Family and Community Pharmacy; g Palliative Care Health Centre; h Dalt San Joan Healthcare Centre, Mahó, Menorca; i Camp Redó Healthcare Centre; j Primary Care Research Unit of Menorca, Balearic Health Services (IB-Salut), Menorca, Balearic Islands, Spain; k Menorca Primary care management, Menorca, Balearic Health Services (IB-Salut); l Son Serra La Vileta Healthcare Centre; m San Agustín Healthcare Centre, Institut d’investigació Sanitària Illes Balears.

**Keywords:** blood pressure control, hypertension, low sodium diet, physical exercise, self-management, self-monitoring

## Abstract

**Introduction:**

High blood pressure is the leading modifiable risk factor for cardiovascular disease, and is associated with high morbidity and mortality and with significant health care costs for individuals and society. However, fewer than half of the patients with hypertension receiving pharmacological treatment have adequate blood pressure control. The main reasons for this are therapeutic inertia, lack of adherence to treatment, and unhealthy lifestyle (i.e., excess dietary fat and salt, sedentary lifestyle, and overweight). Cardiovascular risk and mortality are greater in hypertensive patients who are receiving treatment but have suboptimal control of blood pressure.

**Methods/Design:**

This is a multicentre, parallel, 2-arm, single-blind (outcome assessor), controled, cluster-randomized clinical trial. General practitioners and nurses will be randomly allocated to the intervention group (self-management of antihypertensive medication, self-measurement of blood pressure, hypocaloric and low sodium diet, and physical exercise) or the control group (regular clinical practice). A total of 424 patients in primary care centers who use 2 or more antihypertensive drugs and blood pressure of at least 130/80 during 24-hambulatory blood pressure monitoring will be recruited. The primary outcome is systolic blood pressure at 12 months. The secondary outcomes are blood pressure control (<140/90 mm Hg); quality of life (EuroQol 5D); direct health care costs; adherence to use of antihypertensive medication; and cardiovascular risk (REGICOR and SCORE scales).

**Discussion:**

This trial will be conducted in the primary care setting and will evaluate the impact of a multifactorial intervention consisting of self-management of blood pressure, antihypertensive medications, and lifestyle modifications (hypocaloric and low sodium diet and physical exercise).

## 1 Introduction

Control of hypertension is a major challenge in primary health care, the setting where most such patients are diagnosed and treated. Hypertension has a high prevalence, but only about 45% of patients with hypertension achieve adequate blood pressure (BP) control.^[1,2]^ The successful control of hypertension significantly reduces morbidity and early mortality.^[3]^ Thus, new approaches must be developed that provide better management of high BP.

Hypertension is the main treatable cardiovascular risk factor.^[4,2]^ It causes considerable morbidity and mortality because it increases the risk of myocardial infarction, stroke, kidney damage, and micro- and macro-vascular diseases.^[5]^ The main factors that prevent adequate BP control are therapeutic inertia (use of incorrect dosages and/oran inadequate combination of drugs), poor adherence to treatment,^[6]^ unhealthy lifestyle (smoking, alcohol abuse, excess dietary fat and salt, sedentary habits, and overweight),^[7]^ and prescription of drug that induced hypertension (nonsteroidal anti-inflammatory drugs (NSAIDs), steroids, nasal decongestants, antidepressants, and oral contraceptives).

Many drugs are currently available for reducing BP, and a common strategy is to prescribe a combination of drugs that have different mechanisms of action, with optimization of the dosage of each drug. The optimal dose may be initially considered as 50% or more of the maximum tolerated dose (MTD)^[8]^ and to improve adherence, several drugs should be combined in a single tablet.^[9]^

The most recent guidelines of the American College of Cardiology (ACC)/American Heart Association (AHA)^[10]^ and the European Society of Hypertension (ESH/European Society of Cardiology (ESC)^[9]^ recommend the following nonpharmacological interventions for the control of high BP: restricted intake of salt and alcohol, weight loss, intensification of physical activity with a structured exercise program, use of the Dietary Approaches to Stop Hypertension (DASH) diet, high intake of fruit and vegetables, and potassium supplementation. Physical activity is also useful for the prevention, treatment, and control of hypertension, and several metanalyzes of randomized clinical trials showed that aerobic exercise can reduce BP. However, it is crucial to specify the frequency, intensity, time, and type (FITT) of exercise regimens.^[7,11]^ Other studies support use of the DASH diet for lowering BP. This diet is rich in fruit, vegetables, fiber, and potassium, and has minimal fat and sodium.^[12]^

In addition to pharmacological and nonpharmacological measures, new patient-centered initiatives are now considered essential for the control of hypertension. There is evidence that a patients self-management of a chronic disease can increase motivation, understanding and skills, quality of life, clinical outcomes, and the efficient use of resources.^[13]^ In general, a patients awareness of the need for self-management can make a real difference in the control of a chronic condition.^[14]^ BP self-monitoring (BPSM) might close the gap between the doctors and patients expectations, lead to effective self-management,^[15]^ and enable the patient to play a greater role in managing the condition.

Self-management of BP consists of starting or increasing the dosage of a medicine, based on agreement of the doctor and patient and in accordance with measurements from BPSM. The TASMINH and TASMIN-SR trials,^[16,17]^ which consisted of self-monitoring, self-management, and tele-monitoring of BP measurements, concluded that self-management significantly contributed to the control of hypertension in a primary care setting.

Approximately, half of the patients treated for high BP do not achieve successful control of hypertension. Overall, patients with high BP constitute a heterogeneous group, and include undiagnosed secondary hypertension, undertreated patients, and patients whose treatment was not successful. Patients with uncontrolled hypertension have a higher cardiovascular risk (CVR), and thus require a more comprehensive approach to treatment. Self-management of medications that is based on self-monitoring can play a significant role, and has generated significant interest among researchers.^[18]^

The aim of this trial is to analyze the effectiveness of a comprehensive intervention, consisting of self-management and self-monitoring of hypertension, lifestyle modifications, and optimization of pharmacotherapy, on the control of BP in patients receiving 2 or more antihypertensive drugs.

### 1.1 Aims and hypothesis

#### 1.1.1 Main objective

To analyze the effectiveness of a multifactorial intervention consisting of optimized pharmacotherapy, BP self-monitoring, self-management of antihypertensive medications, and intensive changes in lifestyle, on the reduction of systolic BP at 12 months in patients with uncontrolled hypertension who were initially receiving 2 or more antihypertensive drugs.

#### 1.1.2 Secondary objectives

1. To analyze the effectiveness of the proposed intervention on:
  1. Achieving adequate systolic and diastolic BP values (<140/90 mm Hg) after 12 months.
  2. Quality of life, as measured by a validated Spanish language version of the EuroQol-5D.^[19]^
  3. Reduction of baseline CVR by 10% or more when the baseline risk was low/moderate and by 25% or more when the baseline risk was high/very high.
  4. Adherence to the antihypertensive medication regimen, based on the registry in the prescription section of the electronic medical record of the patient (Medication Possession Ratio).
  5. Body mass index (BMI) and physical activity, as determined by the International Physical activity Questionnaire (IPAQ).^[20]^
  6. Patient safety, in terms of hypertensive crises, number of hypotensive episodes that required an emergency department visit, and adverse effects associated with medications.
  7. Feasibility, acceptability, adherence, and fidelity of the intervention.

## 2 Methods and design

### 2.1 Study design

This is a multicenter, parallel, 2-arm, single-blind, cluster-randomized clinical trial.

The control group will receive regular clinical care according to recommendations for treatment intensification by the Eighth Joint National Committee,^[21]^ with pharmacological and non-pharmacological measures according to the 2018 ESC/ESH Guidelines for the Management of Arterial Hypertension.^[9]^Fig. 1  shows the schedule for enrolment, interventions, and assessments of the study.

**Figure 1:**
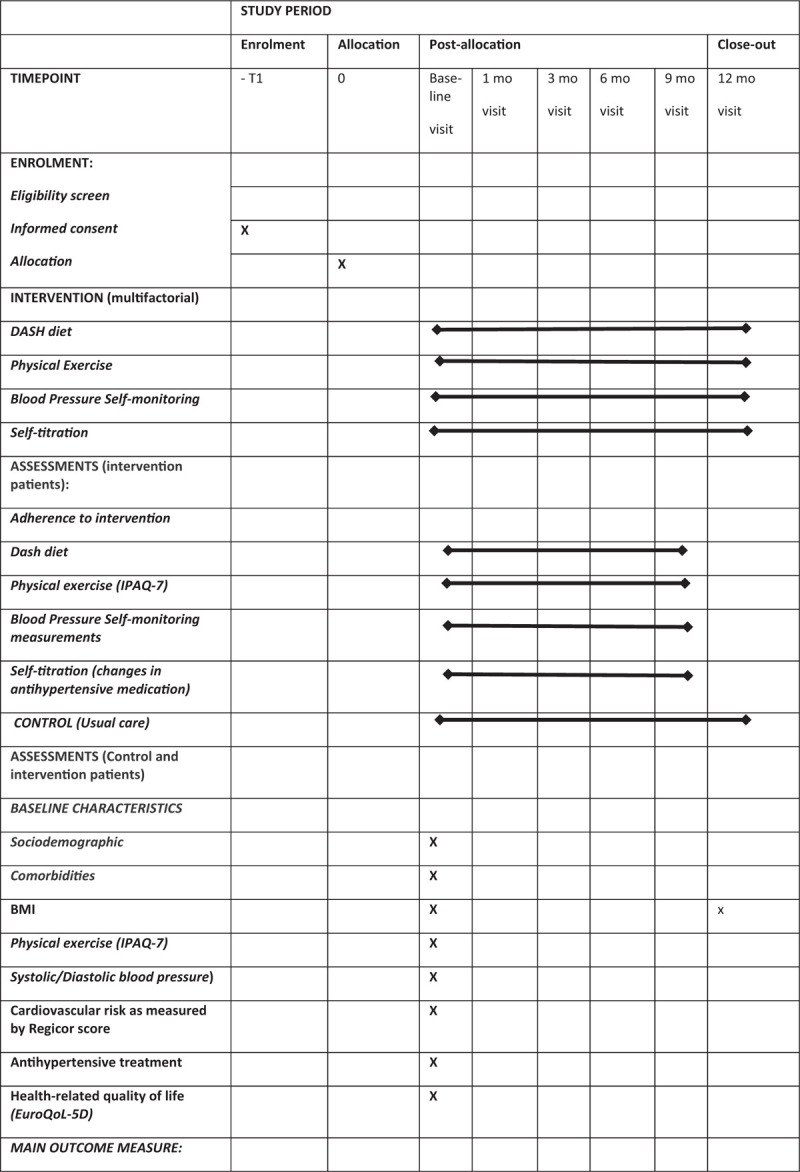
Schedule of enrolment, interventions, and assessments.

**Figure 1 (Continued):**
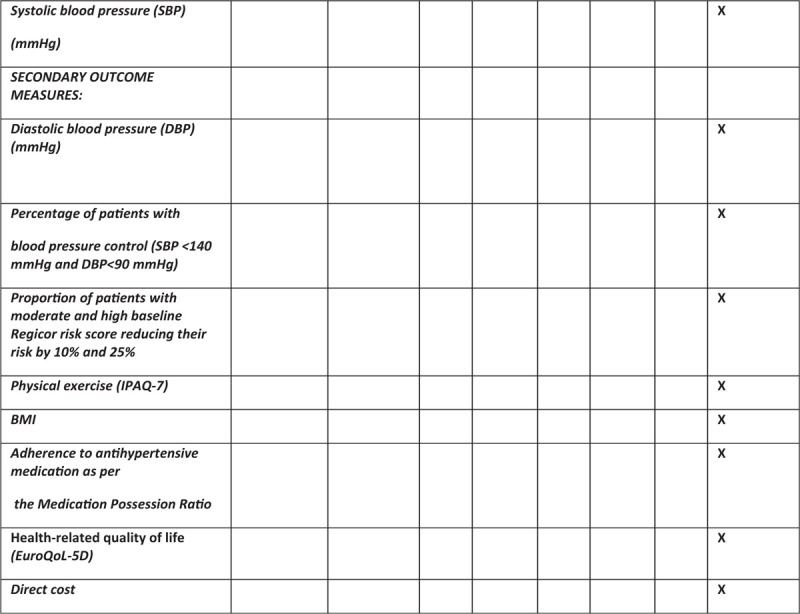
Schedule of enrolment, interventions, and assessments.

### 2.2 Setting and eligibility criteria

#### 2.2.1 Inclusion criteria

1. Hypertensive patients aged 35 to 75.
2. Use of 2 or more antihypertensive drugs.
3. BP of 130/80 mm Hg or more during 24-hambulatory monitoring.
4. Written informed consent.

#### 2.2.2 Exclusion criteria

1. Dialysis or diagnosis of kidney failure.
2. Myocardial infarction, coronary bypass surgery or coronary angioplasty, stroke, peripheral arterial disease, heart failure, advanced liver disease, or atrial fibrillation.
3. Unable or unwilling to sign the informed consent document.
4. Alzheimer disease, advanced dementia or life expectancy less than the follow up duration of the study.
5. Indefinite institutionalization in a nursing home.
6. Long-term treatment with corticosteroids.
7. Secondary hypertension.
8. Impaired kidney function based on albuminuria (>300 mg/24 h)or estimated glomerular filtration rate (<60 ml/min/1.73 m^2^).

### 2.3 Recruitment

GPs and nurses will be recruited in primary care centers of Mallorca and Menorca islands. A list of eligible patients will be obtained from the electronic medical records each GP will systematically assess the eligibility of the patients and invite to participate.

A 24-hABPM examination will be performed before inclusion, and any patient whose BP is 130/80 mmHg or more will be included.

### 2.4 Randomization

GPs will be randomized in a 1:1 ratio to an intervention or control arm after 2 patients were included. Allocation of GPs will be randomized using computer-generated block randomization in blocks of 6. Randomization and allocation concealment will be centralized in a single coordinating center.

### 2.5 Sample size

Sample size calculation, based on a reduction of the SBP of at least 5 mm Hg (SD: 17 mm Hg) between groups, led to an initial estimate of 177 patients per group. Next, correction was applied for correlation among patients within clusters using the inflation factor 1 + *p* (*m* − 1), where *m* is the mean number of observations per cluster and *p* is the intraclass correlation coefficient. Each participating GP will have approximately 10 patients, the expected intraclass correlation coefficient between clusters is 0.04, and the cluster design effect is 1.20. Consequently, the final target sample size is 424 patients (212 in each arm).

### 2.6 Blinding

GPs cannot be blinded to the intervention that seeks to modify their clinical practice, nor the researchers be blinded to the allocation of the GPs. However, the outcome assessor and the data analyst will be blinded to patient allocation.

### 2.7 Interventions

Patients with GPs in the intervention group will receive a multifactorial intervention consisting of changes in diet, physical exercise, self-measurement of BP, and self-titration of antihypertensive medications. An educational session conducted by a nurse will teach patients to use a sphygmomanometer and step counter. A second session will provide information on diet and exercise.

a) Diet: Patients will be asked to follow the DASH diet and to attend a workshop session that will be used to motivate them to adopt this diet. Patients will also learn about reading the labels of food products and consuming adequate portions of each food group.
b) Physical exercise: Moderate aerobic exercise will be prescribed according to the patient's condition. Generally, moderate exercise for 50 to 60 minutes on 5 to 7 days a week will be recommended.^[22]^ If the exercise is walking or running, patients will receive a step counter. Risks associated with physical activity will be evaluated and progressive training will be prescribed, considering heartbeat, distance, steps walked, and time. Study participants will report their subjective degree of fatigue following physical activity.
c) Self-measurement of BP: Patients will receive a BP monitor and will be trained in its use. They will measure BP 3 times in the morning and 3 times in the afternoon during the first and third weeks of each month throughout the 12-month study. Patients will be asked to record their weekly average BP in a table. The patients will also record the use of all medications.
d) Self-titration of antihypertensive medications: Self-adjustment of medications will be performed according to the following algorithm:

1. **For patients with uncontrolled hypertension receiving 2 antihypertensive drugs**
  a) **First Step**: Check that the combination of 2 drugs includes 1 Angiotensin-converting-enzyme (ACE) inhibitor or Angiotensin II Receptor Blockers(ARB) + 1 thiazide (hydrochlorothiazide) or a thiazide-like diuretic (chlorthalidone, indapamide), or 1 CCB (calcium channel blocker). An exception is made if there is a specific reason to use another type of hypertensive drug (such as a beta blocker) due to angina, heart failure, myocardial infarction, control of heart rate, or attempting pregnancy. The dosage of each drug must be at least 50% of the MRD and the drugs should be administered as a single pill when possible.
  b) **Second Step**: Add a third drug to the previous combination, with the resulting combination including and ACE inhibitor (or ARB) + CCB + thiazide or a thiazide-like diuretic. This third drug is added at half the standard dose.
  c) **Third Step**: In patients with no hyperuricemia or diabetes, start increasing the dose of the diuretic up to 100% of the MRD. In patients with hyperuricemia or diabetes, the first medicine to be increased is the ACE inhibitor or ARB.
  d) **Fourth Step**: In patients over 60 years-old, the CCB is the second drug whose dose is increased. In patients under 60 years-old, the ACE inhibitor or ARB is the second drugs whose dose is increased.
  e) **Fifth Step**: The third drug whose dose is increased is the remaining drug (CCB, ACE inhibitor, or ARB).
  f) **Sixth Step**: When 3 drugs were each increased to the optimal dose and hypertension is still uncontrolled, 25 mg of spironolactone is added.
2. **For patients with uncontrolled hypertension receiving 3 antihypertensive drugs**
  a) **First Step**: Check that the 3 drugs are an ACE inhibitor (or ARB) + CCB + thiazide (hydrochlorothiazide) or a thiazide-like diuretic (chlorthalidone, indapamide). An exception is made for patients who receive specific treatments due to other diseases.
  b) **Second Step**: Increase the dose of each drug to at least 50% of the MTD, 1 drug at a time.
  c) **Third Step**: Apply the same rule described for patients receiving 2 antihypertensive drugs. In both cases, each increase of dosage occurs in intervals of 4 to 6 weeks.
  d) Patients whose GPs are in the control group will receive standard care for hypertension.

### 2.8 Measurements

BP will be measured with the patient sitting, using a standardized automated sphygmomanometer (OMROM 7 intelli IT) and after a 5-minrest period. Three readings will be taken 2 minutes apart, and the average of the last 2 will be recorded. The cuff will be adjusted according to the perimeter of the arm and placed on the middle third of the upper arm. The patient will be comfortably seated, without crossing the legs, and with the arm resting on a table and placed at the level of the heart. The patient should be relaxed, have an empty urinary bladder, and have no recent smoking or ingestion of stimulating substances.

### 2.9 Variables

#### 2.9.1 Primary outcome measure

The primary outcome measure is the systolic BP at 12 months, measured according to ESH/ESC guidelines. Prior to measurements, patients will be instructed to not eat or smoke for 30 minutes and to sit quietly with the back supported and feet on the floor for 5 minutes. An oscillometric BP monitor (OMROM 7 intelli IT) will be used for 3 measurements using an appropriately sized cuff and with support of the bare arm.

#### 2.9.2 Secondary outcome measures

The secondary outcomes are:

1. adequate BP control (<140/90 mm Hg) after 12 months;
2. quality of life,^[19]^ measured with the EQ-5D, which has good validity and reliability for individuals with various health conditions and includes 5 dimensions (mobility, self-care, usual activities, pain/discomfort, and anxiety/depression) and a visual analogue scale (VAS) used by patients to report their perceived health status;
3. clinically relevant changes in cardiovascular risk from baseline to 12 months, based on the REGICOR scale,^[23]^ in which differences are defined as changes in the moderate SCORE values and high SCORE values that reduced their risk of a coronary event by 10% and 25%, respectively;
4. adherence to the hypertensive medications, based on the Medication Possession Ratio (MPR) at 12 months;
5. BMI at 6 and 12 months;
6. physical exercise, measured with the International Physical Activity Questionnaire (IPAQ)^[20]^ at baseline and 12 months.

#### 2.9.3 Safety

All serious adverse events, such as hypotension, syncope, bradycardia, electrolyte abnormality, fall, acute kidney injury, or acute renal failure, that are possibly related to the use of antihypertensive drugs will be monitored.

#### 2.9.4 Statistical analysis

Estimated effects will be calculated by comparing the systolic BP (mm Hg) in the intervention and control groups at 12 months. Generalized mixed linear random effect models will be used to account for clustering at the level of GP, with adjustment for the baseline values.

All analyses will be performed on an intention-to-treat (ITT) basis (i.e., all initially randomized patients will be included in the analysis according to group assignment) and results will be reported according to the 2010 CONSORT guidelines.^[24]^ Subgroup analyses of effectiveness will be performed for the following patient groups: with *vs.* without diabetes mellitus; with moderate vs high cardiovascular risk; with vs without adherence to antihypertensive medications; and males vs females. The significance of differences in the baseline characteristics of the control and intervention groups will be analyzed using descriptive analysis and will include calculation of means and/or proportions with confidence intervals, and of standard deviations (to account for clustering).

The relative and absolute risk reduction and number needed to treat (NNT) to prevent one patient from having uncontrolled hypertension (SBP≥140 or DBP ≥90) will be estimated. All estimates will include 95% confidence intervals. The NNT will be calculated as the reciprocal of the difference between the proportion of patients with BP control in the intervention and control arms.

The health economic analysis will be performed by calculating the incremental cost-effectiveness ratio (ICER) at 12 months. Data will be systematically collected on the use of all resources, including inpatient care, consultations with health care providers, use of drugs, and laboratory tests. To measure these effects, EQ-5D scores will be used, and quality-adjusted life years (QALYs) will be determined. The ICER will be calculated as the difference in the mean costs of the 2 groups (C_1_ – C_T_) divided by difference in the mean effects of the 2 groups (E_1_ – E_T_):
 
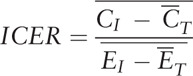


A nonparametric bootstrap procedure will be used to estimate the uncertainty of the estimated ICER. This procedure considers the skewness of cost data, and the covariance of costs and QALYs. To control for possible confounding and to account for clustering, an alternative procedure—Net-Benefit Regression—will also be used. Cost-effectiveness acceptability curves will be presented to show statistical uncertainty. The safety of all interventions will be determined using per-protocol analysis and the Chi-squared test will be used to compare adverse events in the safety population.

## 3 Discussion

Medical self-management requires patients to develop specific preventive, management, and therapeutic skills.^[25]^ Previous studies have evaluated programs that promoted self-management in patients with chronic diseases, such as diabetes, Chronic Obstructive Pulmonary disease (COPD), heart failure, and hypertension,^[26]^ and found that clinical improvement and better disease control occur when patients develop the skills needed to manage their conditions.

Our intervention follows procedures used in previous medical self-management programs.^[27]^ In particular, our intervention includes follow-up and management of signs and symptoms; education regarding drug dosage; changes in lifestyle; and maintaining regular contact with primary health care professionals for shared decision-making.^[28]^

It is particularly important to develop new and effective interventions for patients with uncontrolled hypertension, because these individuals have higher morbidity and mortality rates than patients with controlled hypertension. The participation of patients in management of their own hypertension adds a new dimension to the treatment of this condition. Our intervention, which is comprehensive and implemented in the primary care setting, consists of optimization of pharmacological treatment through self-monitoring and self-management of antihypertensive medications and encouraging patients to adopt healthier lifestyles, including intensification of physical activity and adopting the DASH diet.

This study aims to improve the management of hypertension and addresses pharmacological and non-pharmacological measures that can be used to achieve this goal. The researchers are committed to implementing the intervention, advancing the understanding of this disease, and engaging the patients in the management of their hypertension.

This protocol will provide new data on the use of a comprehensive approach in the primary care setting to control arterial hypertension.

## Acknowledgments

The authors would like to thank Begoña Enseñat, Encarnación Villegas, Paloma Diaz, Adelaida Coll, the Son Espases Hospital Clinical Laboratory and Biobanco IdISBa for organize the extraction, collection and conservation of the blood samples of patients. The authors would also like to thank Sebastià Mesquida for searching the eligible patients and theparticipant GPs of the study for recruitment, collecting data and delivering the interventions.

## Author contributions

FU, FR, JLlC, PLM and ALR are responsible for the original idea and design of the intervention; PLM, ARH, AEM, TRR and MLMS developed the educational material for the study; FUV, JPB, TRR, FRC, PDF, PBF and PLM contacted GPs and nurses in the primary care centres; FUV, PBF, JLC and ALR drafted the manuscript; AL, JLC, and MTQ are responsible for the management of the trial, data analysis and interpretation; BOO will organise the ABPM for the study patients and coordinate with the health centres. All authors (FUV, JLlC, JPB, PBF, PLM, FR, TRR, AEM, MTQ, MLMS, ARH, ALR, BOO and PDF) read the draft critically, made contributions, and approved the final manuscript. FU is the principal investigator, had full access to all data and takes responsibility for the integrity of the data and the accuracy of the data analysis.

Alfonso Leiva orcid: 0000-0001-5306-8533.
